# A Novel Carbon Paste Electrode Modified with N^1^-Hydroxy-N^1^,N^2^-Diphenylbenzamidine for the Electrochemical Determination of Cadmium(II) in Environmental Samples

**DOI:** 10.1155/2022/3426575

**Published:** 2022-09-22

**Authors:** Endale Tesfaye, Bhagwan Singh Chandravanshi, Negussie Negash, Merid Tessema

**Affiliations:** Department of Chemistry, College of Natural and Computational Sciences, Addis Ababa University, P. O. Box 1176, Addis Ababa, Ethiopia

## Abstract

The present study introduces a novel electrode for rapid, highly sensitive, and selective electrochemical sensor for cadmium(II) using 5% N^1^-hydroxy-N^1^,N^2^-diphenylbenzamidine (HDPBA) modified carbon paste electrode (CPE) (HDPBA‒CPE). Surface characterizations and structural analysis of the proposed HDPBA‒CPE were performed using several analytical techniques. The voltammetric measurements of Cd(II) were conducted by cyclic voltammetry (CV) and square wave anodic stripping voltammetry (SWASV). Several experimental conditions such as composition and pH of buffer solutions, HDPBA composition, accumulation potential and time, and other voltammetric conditions were optimized. Cd(II) was preconcentrated on the modified electrode surface for 270 s using Britton Robinson (B-R) buffer (0.1 M, pH 4) at −1.0 V versus Ag/AgCl, followed by electrochemical oxidation of the accumulated Cd(II) in the positive scan of SWASV after a quiet time of 10 s. Under optimized parameters, the proposed method showed a linear range of 0.3–100 nM Cd(II) with a detection limit of 0.032 nM. The fabricated HDPBA-modified carbon paste electrode exhibited excellent sensitivity, selectivity, stability, and reproducibility (with RSD of 3.8%). The developed HDPBA‒CPE was used for the quantification of Cd(II) in tobacco and environmental water samples, and it was found to be applicable for the determination of different types of real samples.

## 1. Introduction

In recent times, environmental contamination has become a worldwide problem because of population growth, agricultural activities, and the rapid development of industries [1]. The continuous release of heavy metals into the surroundings lead to serious problem throughout the world. Environmental monitoring of toxic metal ions is an area of major concern because of their perniciousness and the ability to accumulate in human organs even at trace levels [2]. Therefore, developing a highly selective, sensitive, and fast monitoring methods is very important for the toxic heavy metal ions due to their serious environmental impact and the increased industrial applications [3].

Cd(II) is among the toxic metal ions, which is mostly present in the water around the chemical industries and has become a rising issue of environmental pollution. Cadmium is mostly used in various factories including the plastic industry, coating technology, and battery manufacturing. It is also the major byproduct of Zn smelting work [4]. Food and water samples may have high contents of cadmium(II) when present around cadmium origins. The trace level accumulation of cadmium(II) in the human organs may lead to various health effects such as muscular cramps, renal degradation, erythrocyte destruction, nausea, diarrhea, salivation, and chronic pulmonary problems [5]. Recent research works indicated that the cadmium(II) is a cause for a carcinogenic disease. The United States Environmental Protection Agency set an allowable limit of 5 *μ*g/L Cd(II) in drinking water [4]. Therefore, the fabrication of an accurate analytical technique for the quantification of trace concentrations of cadmium(II) has become of great interest in recent times.

Different methods have been applied for the detection of Cd(II) at trace contents which include atomic fluorescence spectrometry [6], graphite furnace atomic absorption spectrometry (GFAAS) [7], inductively coupled plasma-mass spectrometry (ICP-MS) [8], high performance liquid chromatography (HPLC) [9], inductively coupled plasma-atomic emission spectroscopy (ICP-AES) [10], and X-ray fluorescence [11]. The aforementioned analytical methods are very reliable and accurate for the quantification of Cd(II). But these analytical techniques have major limitations including their expensiveness, tediousness, and time-consuming nature. Furthermore, they are not appropriate for in-situ analysis and require various complicated steps and skills [12].

Instead of the aforementioned methods, the voltammetric methods have been used as the most versatile and suitable techniques for the detection of heavy metals including Cd(II), because of their high sensitivity, high selectivity, fast analysis, simplicity, portability, low cost, easy maintenance, and the ability to conduct speciation determination [13].

Nowadays, chemically modified carbon paste electrodes (CMCPEs) have been applied as selective and suitable tools for the analysis of metal ions because of their easily regenerated surface, cost-effectiveness, simple modification, low background current, high sensitivity, and selectivity of electrochemical methods. The working mechanisms of electrodes depend on the nature and characteristics of the modifiers applied [14]. Several types of modifiers including organic ligands, polymers, ion exchangers, and nanomaterials are usually used in the modification of carbon electrodes [15].

Ligands modified carbon paste electrodes (CPEs) have been applied for the electrochemical determination of toxic metal ions because of their specific complexing ability with metal ions. Most of the metal-chelating ligands have acidic groups, such as, −COOH, ‒SH, ‒OH, and neutral donating groups which can form stable complexes with metal ions [16].

Several research works have been conducted on the quantification of cadmium and other metal ions using CPEs modified with chelating ligands such as carbamoyl phosphonic acid-mesoporous silica modified CPE for Cd(II), Pb(II), and Cu(II) [17], 4-((1H-1,2,4-triazol-3-ylimino)methyl) phenol (L)-multiwalled carbon nanotubes composite modified carbon paste ionic liquid electrode for Cd(II) [18], 3,6-bis(2-(2-sulfanyl-ethylimino)-methyl)-4-(4-nitro-phenylazo)-phenol-pyridazine-SiO_2_ modified CPE for Cd(II) and Cu(II) [19], N,N-bis(3-(2-thenyldiamine) propyl) piperazine silica nanoparticles modified CPE for Cd(II), Cu(II), and Hg(II) [20], N-*p*-chlorophenylcinnamohydroxamic acid for Cd(II) [21], and Hg(II) [22]. Most of the previous research studies for the detection of Cd(II) have used many modifiers which are costly, tedious, and time-consuming.

**Figure 1:**
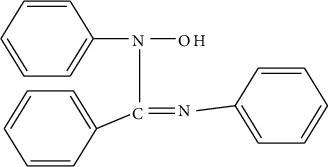
Structure of N^1^-hydroxy-N^1^,N^2^-diphenylbenzamidine (HDPBA).

HDPBA (Figure 1) is one of the groups of ligands which were employed for the spectroscopic determination of various metal ions including Mo(V), Ti(IV), Mn(VII), V(V), Co(II), and Fe(III) [23–29]. HDPBA has also been used for the voltammetric determination of Hg(II) and Pb(II) [30, 31]. However, HDPBA has not been used for the voltammetric detection of cadmium(II). The N and O atoms in the HDPBA provide the modifier with a high affinity towards metal ions to form stable complexes [32].

This study was aimed at fabricating a new HDPBA modified carbon paste electrode (HDPBA-CPE) for sensitive electrochemical determination of Cd(II) using SWASV. Unlike the previous reported electrodes, in this paper, a single modifier (HDPBA) is used for the modification of CPE which is less costly and not time consuming. HDPBA was synthesized by the condensation of N-phenylhydroxylamine with N-phenylbenzimidoyl chloride at 0°C in ether which is an easy and cost-effective preparation method. The modified electrode exhibited the characteristics of cost-effectiveness, simple preparation, excellent stability, and easy renewable ability. The fabricated sensor has also a higher sensing ability for Cd(II) than electrodes with one and more modifiers. The developed electrode was effectively used for the quantification of trace levels of Cd(II) in various types of water samples and tobacco samples.

## 2. Materials and Methods

### 2.1. Chemicals and Instruments

Graphite powder (spectroscopic grade, BDH Laboratory Supplies Poole, England), paraffin oil (Merck, Germany), and cadmium standard solution (Sigma-Aldrich, USA) were employed in this study. Different concentrations of Britton Robinson (B-R) buffer were prepared using phosphoric acid (Riedel-de Haën Chemicals, Germany), acetic acid (Sigma-Aldrich, USA), and boric acid (Carlo Erba Reagents, Cornaredo, Italy) using distilled water. The B-R solution was adjusted to different pH values with an addition of a 0.1 M sodium hydroxide solution (Avonchem, United Kingdom). Sodium acetate, ammonium chloride, sodium perchlorate, hydrochloric acid, sodium phosphate (BDH Chemicals Ltd, England), and sodium citrate/citric acid were applied for the preparation of working standard solutions in optimization studies. Reagent grades sodium sulfate, sodium chloride, magnesium chloride (AnalaR, Hopkin & Williams Ltd, England), calcium nitrate (Aldrich Chemical Co. Ltd Gillingham Dorset, England), potassium nitrate (BDH Chemicals Ltd Poole, England), cupric nitrate (AnalaR, Hopkin & Williams Ltd, England), lead nitrate (BDH Chemicals Ltd Poole, England), mercury standard (Aldrich Chemical Company, Inc, USA), cobalt nitrate (Riedel deHaen, Honeywell Specialty Chemicals Seelze GmbH, Germany), nickel nitrate (BDH Chemicals Ltd Poole, England), iron(III) nitrate (Riedel deHaen, Honeywell Specialty Chemicals Seelze GmbH, Germany), and ammonium chloride (Merck Reagent, Germany) were used for interference study. Hydrogen peroxide (30%) and nitric acid (68%) were used for the digestion of real samples. Argon (99.999% Merck-Schuchardt) was used for the deaeration of test solutions. HDPBA was synthesized by the reaction of N-phenylhydroxylamine with N-phenylbenzimidoyl chloride at 0°C in ether [26].

The electrodes morphology characterizations were studied by a scanning electron microscope (Cx-200 COXEM, Korea). The Fourier transform infrared (FTIR) spectrometer (PerkinElmer, Spectrum 100, USA) was used for the recording of the FTIR spectra. UV-Vis analyses were done using a UV/Vis/NIR instrument (PerkinElmer Lambda 950, USA). The electrochemical measurements were conducted using the CHI 840C electrochemical analyzer (CHI instruments, USA). Three electrodes system with modified or unmodified CPE as a working electrode, Ag/AgCl as a reference electrode, and Pt-wire as a counter electrode were used in all the voltammetric determinations of the study. A 20 mL cell was used as a solution container. A pH meter (sensION, China), magnetic stirrer, and a stop clock were applied for pH measurements of the solutions, stirring of the solutions, and time measurement, respectively. Atomic spectrometric determination of Cd(II) was conducted using a graphite furnace atomic absorption spectrometer (GFAAS) (Agilent 280Z AA Zeeman, GTA120, USA).

### 2.2. Electrodes Preparation

Modified CPE was made by mixing of 0.05 g of the modifier (HDPBA), 0.95 g graphite powder (spectroscopic grade, BDH Laboratory Supplies Poole, England), and 0.36 mL paraffin oil (Uvasol Merck, Germany) using a mortar and pestle. In the mixture, the paraffin oil does not dissolve the modifier (HDPBA). The paraffin oil was used to adhere the powder and the HDPBA to form a uniform paste. Hence, the HDPBA still remained solid in the carbon paste. The mixture was ground for 20 min and packed into an electrode prepared from plastic syringe (1 mL) with a 3 mm outer diameter, using a Cu wire connected to measurement instrument. The bare CPE was made in an identical manner, without the addition of the modifier (HDPBA). The renewal of the electrode was performed by removing a small portion of the electrode surface and replacing it with a fresh paste.

### 2.3. Voltammetric Determinations

The voltammetric determinations were performed using CV and SWASV by applying the following steps: (a) accumulation step: In this step, the accumulation solution, 0.1 M B-R buffer (pH 4) was first added into the cell, and a predetermined concentration of Cd(II) was added to it. After immersion of the electrodes into the cell, Cd(II) was preconcentrated at the electrodes surface with an applied potential of −1.00 V for a known time under stirring solution. After preconcentration, stirring was stopped and a 10 s rest time was permitted to make the solution stable and minimize the background current. (b) Stripping step: The voltammograms of Cd(II) were recorded in the positive potential scan from −1.2 to −0.2 V versus Ag/AgCl. During each determination, the electrode was renewed using 4 to 5 runs/scans in B-R solution (0.1 M). Fresh buffer solution was applied in each measurement for the prevention of buildup of potential in the cell. The effect of deaeration on the peak currents of Cd(II) was studied by purging the test solution with argon with different times (0, 5, 8, and 10 min).

### 2.4. Real Samples Analysis

The proposed electrode was employed for the quantification of Cd(II) in tobacco samples and five types of environmental water samples including industrial wastewater, bottled, well, and stream water samples under optimized experimental parameters. Filtration was performed for the water samples before the determination to avoid solid particles. Their pH values were set to 4 and added to the 20 mL·cell, accumulated and determined by voltammetric measurements. A known concentration of Cd(II) was added into each water sample for recovery studies. Furthermore, Cd(II) in the water samples was also determined by the GFAAS method.

A tobacco sample was collected from ten local cigarettes and dried in an oven at 105°C and ground. For the determination of cadmium(II), 500 mg of powdered samples were added into the digestion vessel, and 5.0 mL of H_2_O_2_ (30%) plus 3.0 mL of concentrated HNO_3_ were added. The vessel was closed and placed in the carousel of the oven and kept for about eight minutes. Then the evaporation of the digest was done to near dryness. Finally, HNO_3_ was added for the dissolution of the residue, and voltammetric measurement was done after filtration and pH adjustment of the solution [33].

## 3. Results and Discussions

### 3.1. Characterization of the Developed Electrodes

#### 3.1.1. Spectroscopic Studies and Surface Characterization

The spectral analysis of the modifier (HDPBA) and morphological characterization of unmodified CPE and HDPBA‒CPE were performed using the FT-IR, UV-Vis and SEM techniques. Chemical characterization of the modifier (HDPBA) and unmodified and modified CPEs was done by using Fourier transform infrared (FTIR) analysis. The FTIR characterizations of the HDPBA-modified graphite and unmodified graphite were done by mixing the HDPBA and the graphite powder in a mortar and pestle without paraffin oil addition. First, the HDPBA and the graphite powder were mixed properly in a mortar and pestle. Then, after, prior to spectra acquisition, about one mg of graphite powder (for unmodified paste), while one mg of the mixture of graphite powder and the modifier, HDPBA (for modified paste), was mixed with approximately 100 mg of KBr powder separately using a mortar and pestle. The paste was then compressed to form a pellet. A KBr pellet was also used as the background reference spectrum. The FTIR spectra were recorded using a PerkinElmer with a spectral resolution of 1 cm^−1^ between the wave number 4500 and 500 cm^*−*1^.

**Figure 2:**
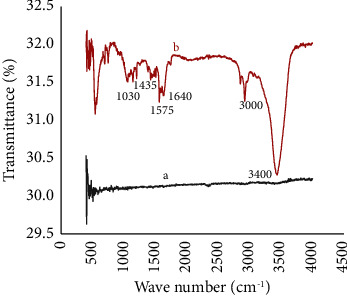
FT-IR spectra of unmodified graphite (a) and HDPBA modified graphite (b).

FTIR spectra of the modifier (HDPBA) and modified and unmodified CPEs were run within the spectral range of 4500–500 cm^−1^. As can be seen in Figure 2, no important bands were found in unmodified graphite (spectrum a). But HDPBA‒graphite mixture (spectrum b) exhibited bands at 1030, 1435, 1575, 1640, 3000, and 3400 cm^−1^ characteristics to the stretching of N−O, C−N, C = C_(aromatic)_, C = N, C−H_(aromatic, stretching)_, and O−H bonds, respectively, in the ligand chemical structure. The results are consistent with other reported literature [16, 23]. The spectral data proved the incorporation of HDPBA in the modified electrode.

**Figure 3:**
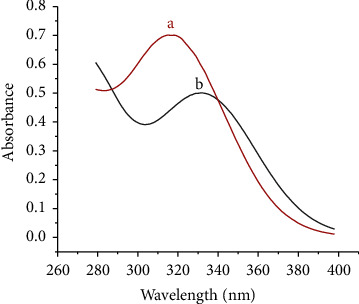
UV spectra of 60 *µ*M HDPBA in ethanol (a) in the absence of Cd(II) and (b) in the presence of Cd(II).

The UV spectra of the modifier were recorded in ethanol. The free modifier (HDPBA) showed a band at 317 nm (Figure 3, curve a) which is assigned to n−*π*^*∗*^ transition of the C=N group and which is in agreement with the results of previous studies [16, 23, 24]. However, after incorporation of Cd(II) into the modifier, the spectra resulted in a bathochromic shift (*λ*_max (ethanol)_ = 334 nm) (Figure 3, curve b). The spectrum shift after the addition of the analyte to the ligand gives evidence for the complex formation between Cd(II) and ligand.

**Figure 4:**
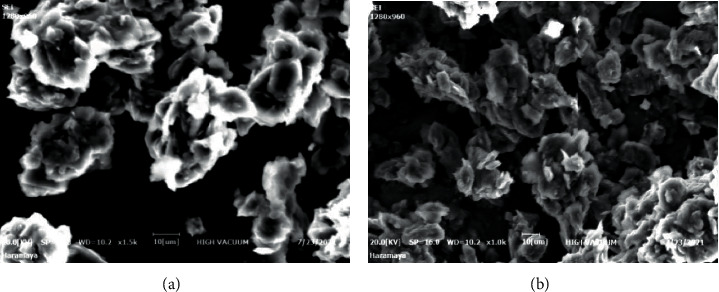
SEM images of (a) unmodified CPE and (b) HDPBA‒CPE. Voltage: 20 keV, magnification: 1000×.

The surface characteristics of the prepared electrodes were studied by SEM. The SEM images exhibited an important difference between the surface morphologies of HDPBA modified CPE and unmodified CPE. The unmodified surface was prevailed by uniformly distributed and homogenous surface with small pores (Figure 4(a)); while after modification, the numbers and pore sizes were much larger, and their distribution on the electrode surface became uneven because of the addition of the ligand into the paste (Figure 4(b)) making it favorable for the adsorption of Cd(II) on the electrode surface resulting in better sensing.

#### 3.1.2. Electrochemical Characterization

The electrochemical behaviors of the unmodified carbon paste electrode and HDPBA‒CPE were studied using CV and electrochemical impendence spectroscopy (EIS) characterizations in K_3_[Fe(CN)_6_]/KCl solution.

**Figure 5:**
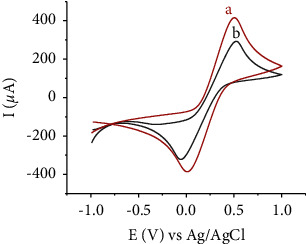
CVs of K_3_[Fe(CN)_6_] at unmodified CPE (a) and HDPBA‒CPE (b); scan rate: 0.1 V/s.

The cyclic voltammograms (CVs) of ferricyanide for both the unmodified and modified electrodes are shown in Figure 5. Intense redox peaks appeared for the unmodified carbon paste electrode (peak a). However, with the HDPBA modified electrode, the peak currents became smaller, and the potential values changed noticeably (peak b). This indicates the ferric cyanide ions repel the lone pair electrons of−OH and −N in HDPBA, consequently the ligand act as blocking layer for mass and electron exchange to obstruct the movement of [Fe(CN)_6_]^3−^ towards the electrode surface [13,34–37], which evidently confirm HDPBA is effectively incorporated on the carbon paste electrode surface.

**Figure 6:**
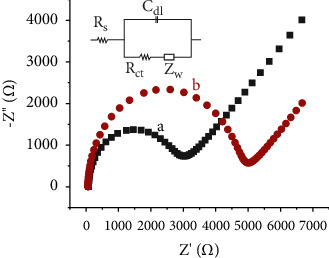
Electrochemical impendence spectroscopy plots of the unmodified CPE (a) and HDPBA‒CPE (b) in 5 mM K_3_[Fe(CN)_6_]/KCl (0.1 M) solution.

EIS is an important tool for investigating the electrochemical characteristics of an electrode's surface. The main parameters in EIS are charge transfer resistance (R_ct_), double layer capacitance (*C_dI_*), electrolyte resistance (Rs), and impedance (*Z*_w_ (W)). Among the mentioned parameters, charge transfer resistance (R_ct_) is an important parameter that can be used to explain the electrode interface properties. The semicircle in the impedance plot corresponds to R_ct_. R_ct_ controls the kinetics of the reaction probe at the electrode surface. In the present study, for electrochemical impendence spectroscopy (EIS) measurements, EIS plots of the developed electrodes were done in 5 mM K_3_[Fe(CN)_6_]/KCl solution in a frequency range of 0.1–100000 Hz with an amplitude of 0.005 V at a potential of 0 V versus Ag/AgCl. From Figure 6, the HDPBA‒CPE has high charge transfer resistance value (*R*_ct_ = 3822 Ω) compared to the unmodified CPE (*R*_ct_ = 2053 Ω). Furthermore, as recorded from the circuit on the instrument, the *C_dI_*, Rs and Z_W_ values for HDPBA‒CPE are 3.18 × 10^−7^ S, 272.4 Ω, and 8.76 × 10^−3^ Ω, respectively; whereas the *C_dI_*, Rs and *Z*_W_ values for unmodified CPE are 9.32 × 10^−6^ S, 105.7 Ω, and 2.76 × 10^−4^ Ω, respectively. These results clearly indicate that the unmodified CPE has been successfully modified with the HDPBA. The EIS results are consistent with the CV results (Figure 5). The results of CVs and EIS for K_3_[Fe(CN)_6_] confirm the modified and unmodified electrodes have significant differences in their surface electrochemical properties, which prove the successful immobilization of the modifier (HDPBA) in the carbon paste electrode.

**Figure 7:**
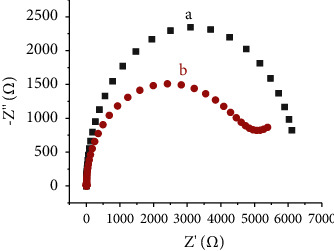
EIS plots of the unmodified CPE (a) and HDPBA modified CPE (b) in 0.1 M Britton Robinson (B-R) solution having 100 *µ*M Cd(II). Potential: 0 V; frequency: 0.1–100000 Hz; amplitude: 0.005 V.

Furthermore, EIS spectra for modified and unmodified electrodes were also recorded in B-R solution (0.1 M) containing 100 *µ*M Cd(II). EIS plots of the HDPBA modified and unmodified CPEs in B-R buffer containing 100 *µ*M Cd(II) are shown in Figure 7. The unmodified electrode has a higher R_ct_ and semicircle diameter than the HDPBA modified CPE. This shows the presence of better affinity between HDPBA and Cd(II). This can be explained by the fact that the electrons of −N and −OH in HDPBA attract the Cd(II) (from the solution), and hence, it leads faster electron transfer within the HDPBA‒CPE [13, 34–37].

### 3.2. Voltammetric Determination of Cd(II)

**Figure 8:**
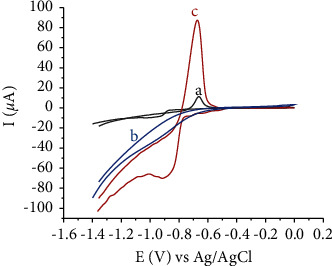
CVs of (a) unmodified CPE with accumulation of Cd(II), (b) HDPBA‒CPE without accumulation of Cd(II) and (c) HDPBA‒CPE with accumulation of Cd(II) in Britton Robinson (B-R) buffer (pH 4) at a potential of −1.0 V. Accumulation time: 270 s; Cd(II) concentration: 100 nM; scan rate: 0.1 V/s.

Figure 8 depicts the CVs of Cd(II) at unmodified and HDPBA modified CPEs in Britton Robinson (B-R) (0.1 M, pH 4). No peaks appeared within the given potential range on the unmodified electrode (not shown) and HDPBA‒CPE (curve b) without the accumulation of Cd(II). After the accumulation of Cd(II), the unmodified electrode (curve a) exhibited a small anodic peak at −0.661 V and a weak and broad cathodic peak at −0.970 V, whereas the HDPBA‒CPE (curve c) shows a sharp anodic peak at −0.673 V and a strong cathodic peak at −0.925 V. The peak intensity increment and negative potential shift at HDPBA modified CPE are due to the complex formation of Cd(II) with HDPBA. The results indicate that unmodified CPE is not able to promote an effective interaction between Cd(II) and the electrode surface. However, HDPBA has an effective interaction with Cd(II). Since Cd(II) is a Lewis acid, it has a preference for complexing/bond formation with ligand (HDPBA) that is Lewis base and has electronegative donor atoms (nitrogen and oxygen) (Figure 1). This results in the higher preconcentration of Cd(II) on the HDPBA‒CPE surface and hence it makes larger current responses. The anodic peak current of Cd(II) is significantly larger and sharper than the corresponding cathodic peak current. Thus, the anodic peak of Cd(II) was studied by SWASV for analytical applications.

**Figure 9:**
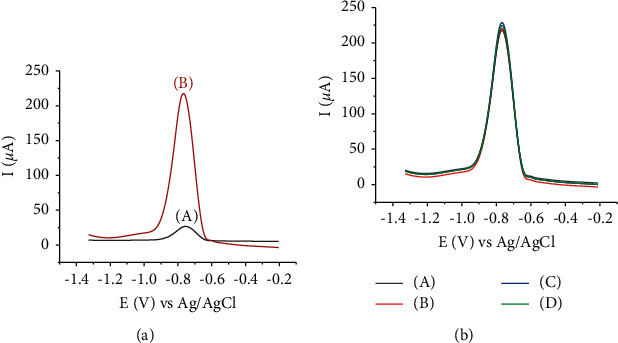
(a): SWASVs of 100 nM Cd(II) in Britton Robinson (B-R) buffer (0.1 M, pH 4) at (A) unmodified CPE and (B) HDPBA‒CPE. Accumulation time: 270 s; potential −1.0 V; amplitude: 100 mV; frequency: 80 Hz; step potential: 6 mV. (b): Square wave voltammograms of 100 nM Cd(II) at HDPBA modified CPE in 0.1 M B-R solution (pH 4) (A) nondeaerated solution (0 min), (B) deaerated solution for 5 min, (C) dearated solution for 8 min, and (D) dearated solution for 10 min.

Figure 9(a) shows the square wave anodic stripping voltammograms (SWASVs) of Cd(II) at the developed electrodes. The anodic peak current of Cd(II) at the HDPBA‒CPE is enhanced significantly compared to that of the unmodified one. Furthermore, the HDPBA‒CPE shows a shift in the peak potential to the negative values (−0.768 V for the HDPBA-modified electrode and −0.756 V for the unmodified CPE) which indicates the formation of a complex between the ligand (HDPBA) and the Cd(II). This is due to the fact that the electrons of −OH and −N groups in HDPBA (Figure 1) attract the Cd(II) and form stable complexes on the HDPBA‒CPE surface, which leads to the accumulation of a higher concentration of Cd(II) and hence finally gives higher current responses. Thus, the use of the HDPBA as a modifier is worthwhile since it highly enhances the analytical peak signal of the electrode.

The influence of deaeration on the current response of Cd(II) was studied by purging the test solution with argon at different times (0, 5, 8, and 10 min). The results are shown in Figure 9(b). No significant difference was found between the peak current of non-deaerated and deaerated solutions. The RSD of the responses among nondeaerated and deaerated solutions with different times (0, 5, 8, and 10 min) was found to be 1.80%. The relative error in the peak current between nondeaerated and deaerated solutions was 3.74%. This indicates the results obtained from both solutions are reproducible and deaerating the electrolyte solution has no substantial effect on the peak currents of Cd(II). Since reproducible and stable results were obtained without deaerating the buffer solution, deaeration (which is an additional step) was not required in this study.

Based on the results found and similar research reported [15, 19, 21, 22], the suggested reaction mechanisms for the redox of Cd(II) on the surface of modified CPE (MCPE) is given below:

Preconcentration step

(Cd^2+^) solution + MCPE ⟶ (Cd^2+^−MCPE) complex

(Cd^2+^−MCPE) complex + 2e^−^ ⟶ (Cd−MCPE) complex

Stripping step

(Cd−MCPE) complex − 2e^−^ ⟶ (Cd^2+^) solution/surface + MCPE

### 3.3. Optimizations of the Experimental Parameters

#### 3.3.1. Effect of Modifier Content

The effect of modifier content in the paste on the peak current of Cd(II) was studied by preparing six separate electrodes made from 2.0%, 3.5%, 5.0%, 7.5%, 10.0%, and 12.5% (w/w) modifiers in the carbon pastes under identical preparation conditions. As presented in Table 1, the highest peak current of Cd(II) was found at the modifier composition of 5.0%. The increase in the peak current response up to 5% modifier composition is due to the fact that the presence of sufficient amount of HDPBA on the electrode surface results in accumulation of a larger amount of analyte ions (Cd(II)). This is because of the complexation of Cd(II) with the HDPBA at the electrode surface. The low peak current at higher modifier content (>5.0%) can be explained by the reduction in the amount of conductive carbon powders within the paste which results in the enhancement in the background current and the decrease in electrode conductivity. While at lower modifier content (<5.0% of HDPBA), there could be a lower extent of complexation between the modifier and Cd(II) and hence it leads to a decrease in the peak current response of Cd(II). Thus, 5.0% HDPBA modified electrode was used for all further experiments.

#### 3.3.2. Effect of Supporting Electrolyte Solutions and pH

The effect of supporting electrolyte solutions on the peak current of Cd(II) was determined by applying different buffers including CH_3_COONa, NH_4_Cl, NaClO_4_, HCl, Na_2_HPO_4_/NaH_2_PO_4_, Na_3_C_6_H_5_O_7_, and Britton Robinson (B-R) (0.1 M). Compared to others, the highest peak current was found in the Britton Robinson (B-R) buffer. This may be due to the ionic strength and buffering capacity differences between the solutions. Therefore, Britton Robinson (B-R) buffer was selected as a suitable supporting electrolyte solution in the study.

**Figure 10:**
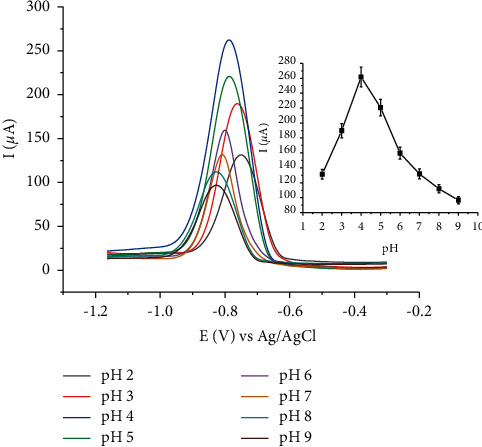
Effect of pH of Britton Robinson (B-R) solution on the peak current of Cd(II); Cd(II) concentration: 100 nM. Accumulation time: 270 s; accumulation potential −1.0 V; amplitude: 100 mV, frequency: 80 Hz; and step potential 6 mV.

The pH effect was studied by varying the pH of the B-R solution between 2 and 9. As shown in Figure 10, the highest voltammetric peak was obtained at pH 4. The low peak currents Cd(II) at extremely low pHs (<4) may be due to the competition of the protons in the solution with Cd(II) for attachment to the modifier, HDPBA. Furthermore, at excessively low pH values, the dissolution and leakage of the modifier (HDPBA) may gradually occur in the acidic medium, which results in the loss of the capability of the HDPBA to immobilizing Cd(II). The hydrolysis of Cd(II) might be carried out at higher pHs (pH > 4), which leads to a decrease in the peak current of Cd(II) due to the lack of preconcentrated Cd(II) on the modified electrode surface [33].

#### 3.3.3. Effect of Accumulation Potential

**Figure 11:**
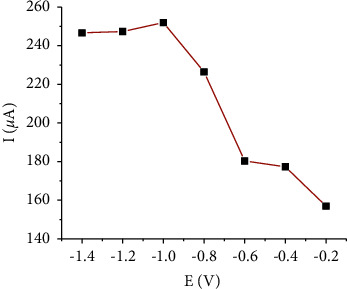
Accumulation potential effect on the voltammetric current of 100 nM Cd(II). Supporting electrolyte solution: B-R buffer of pH 4; accumulation time: 270 s; amplitude: 100 mV; frequency: 80 Hz; and step potential: 6 mV.

The effect of preconcentration potential was determined by changing the potential values between −0.2 V and −1.4 V. As depicted in Figure 11, the peak currents of Cd(II) improved significantly with negative values up to −1.0 V and the highest peak current was found at −1.0 V. However, after −1.0 V, the current responses could not be obviously increased; therefore, −1.0 V was chosen for this study. A decrease in the current response at more positive potentials can be explained by the fact that Cd(II)) may not be effectively reduced because of the lack of negative potential. On the other hand, at extremely negative accumulation potentials, a slight reduction of peak currents might be due to impurities that could be easily reduced with the analyte. In addition, at more negative potentials, the evolution of the hydrogen bubbles may affect the deposition of metal at the electrode surface, which results in the decreased current response [38].

#### 3.3.4. Effect of Accumulation Time

**Figure 12:**
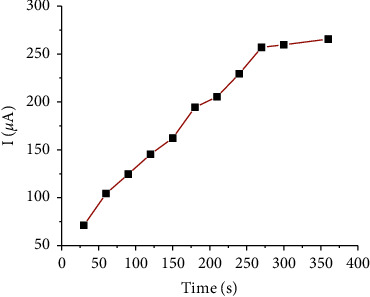
Accumulation time effect on the current signals of 100 nM Cd(II). Supporting electrolyte solution: B-R solution of pH 4; accumulation potential: −1.0 V; amplitude: 100 mV; frequency: 80 Hz; and step potential: 6 mV.

It was determined by varying the time between 30 s and 360 s. As indicated in Figure 12, the peak current increases quickly up to 270 s and then slowly increases after 270 s, which shows a significant effect of preconcentration time on the detection sensitivity of Cd(II). The gradual increase of current signals of Cd(II) at higher accumulation times is attributed to the accomplishment of saturation of the surface of the modified electrode with accumulated Cd(II). Thus, 270 s was selected as the optimum preconcentration time for the experiments performed in the present study.

#### 3.3.5. Effect of Scan Rate

**Figure 13:**
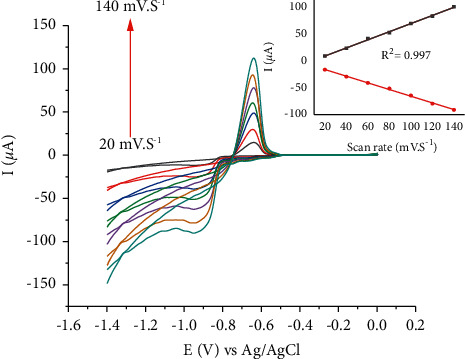
Cyclic voltammograms of 100 nM Cd(II) in 0.1 M B-R buffer of pH 4 at HDPBA-CPE with different scan rates (bottom to top: 20, 40, 60, 80, 100, 120, and 140 mV·S^−1^). Inset: plot for the peak currents of Cd(II) *vs ν*.

For the investigation of the types of interaction between the Cd(II) and the electrode, the scan rate effect was done by varying the values from 20 mV·s^−1^ to 140 mV·s^−1^.  Figure 13 shows the redox peaks at different scan rates (*ν*). The peak currents were linearly proportional to scan rate; the current responses increased with an increase in *ν* without potential shift. This indicates that Cd(II) undergoes a nondiffusion meaning surface adsorption controlled mechanism at the modified carbon paste electrode.

#### 3.3.6. Effect of Instrumental Parameters

The effect of frequency on the peak current of Cd(II) was evaluated by changing its values between 20 and 120 Hz. A frequency of 80 Hz was selected as the optimum value based on the peak shape and symmetry. The effect of amplitude was varied from 20 to 150 mV. A 100 mV was selected for subsequent measurements. Similarly, the step potential effect on the current responses was studied by changing the potential values between 2 mV and 12 mV. A 6 mV was selected as the optimum value.

### 3.4. Analytical Performance

**Figure 14:**
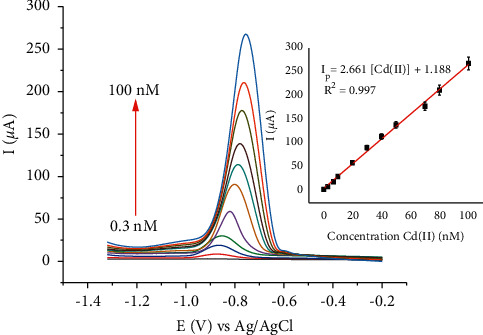
SWASVs of various concentrations (bottom to top: 0.3, 3, 7, 10, 20, 30, 40, 50, 70, 80, and 100 nM) of Cd(II). Inset: calibration plot of peak currents versus Cd(II) concentrations. Buffer: Britton Robinson (B-R) (0.1 M, pH 4); accumulation potential: −1.0 V; accumulation time: 270 s; frequency: 80 Hz; pulse amplitude: 100 mV; and step potential: 6 mV.

To determine the relationship between the anodic peak current and Cd(II) concentration and to construct calibration curves for the electrode, square wave voltammograms were recorded in B-R buffer solution at pH 4 under optimized experimental parameters.  Figure 14 shows the square wave voltammograms and calibration plot for Cd(II) in the linear range of 0.3 to 100 nM. A calibration equation: *I*_p_ = 2.661 [Cd(II)] + 1.188 with a *R*^2^ value of 0.997 was found in the given linear range. The limit of detection calculated as LOD =  3*s*/*m* was found to be 0.032 nM, where LOD, *S,* and *m* are the detection limit, standard deviation of 0.3 nM of Cd(II), and the slope of the calibration, respectively. The *s* was calculated by 8 times measurements of 0.3 nM of Cd(II) peak signals. The calibration results confirm that the developed HDPBA/CPE showed an excellent sensing performance/affinity towards the detection of trace Cd(II).

The repeatability of the fabricated electrode was investigated by seven repeated determinations of 50 nM Cd(II) using a similar electrode in identical conditions. RSD value for seven repeated measurements of 50 nM Cd(II) was obtained to be 2.6%. The reproducibility for 50 nM of Cd(II) solution was also performed by applying five separate electrodes, which were made in an identical manner. The RSD of the results found between the five electrodes was 3.8%, which indicates the excellent precision of the proposed sensor. In addition, the stability of the modified electrode was performed by recording the current signals of 100 nM Cd(II) for one month. After one month, the peak current response of 100 nM Cd(II) only decreased by 4.2%. The excellent stability and reproducibility of the modified electrode revealed its applicability for the quantification of Cd(II) in real samples.

### 3.5. Selectivity of the Modified Electrode

The effect of foreign ions on the current response of Cd(II) was studied by the addition of different concentrations of various ions into 5.0 × 10^−8^ M of Cd(II) solution during the accumulation step. The interfering ions were determined in five different groups. The effect of detected interferences on the current signal of Cd(II) is shown in Table 2. As shown in the table, the detected ions had a negligible interference effect on the determination of Cd(II). There was not a significant effect on the current response of Cd(II) with the addition of 7 to 100 fold molar excess of five groups of the interfering ions in 5.0 × 10^−8^ M of Cd(II) solution. This revealed that the developed electrode has excellent selectivity and can be used for the detection of Cd(II) in real samples among other ions.

### 3.6. Comparison of the Proposed Electrode's Analytical Performance with Reported Electrodes

The performance of the present electrode was compared with several previous electrodes for the determination of Cd(II) described in the literature. As shown in Table 3, the LOD obtained in the present study is much better than other electrodes except one electrode [44]. However, this electrode has a relatively long extraction/preconcentration time (15 min) compared to the present study (4.5 min). A fast preconcentration time used in the present method enables faster measurements. Furthermore, the present method has better and comparable linear range and accumulation/preconcentration time to most of the reported electrodes. The results indicate the excellent analytical performance of the proposed method and its applicability for the determination of Cd(II) at trace concentrations.

### 3.7. Novelty of the Study

In this study, a new modified CPE has been fabricated using laboratory prepared ligand, HDPBA, as a modifier for the detection of Cd(II) at low concentrations using the SWASV technique. The developed modified electrode using a single modifier has achieved a better sensing ability with a lower limit of detection than other reported electrodes having more than one modifiers which made them more costly and time-consuming during electrode preparation. Moreover, the fabricated electrode exhibited many attractive features including cost-effectiveness, simple preparation, easily regenerated surface, and fast analysis. It also has a great potential for the analysis of various types of real samples without significant matrix effects due to its high sensitivity and selectivity and excellent reproducibility.

### 3.8. Analytical Applications

To determine the suitability and feasibility of the developed electrode for real samples, the fabricated electrode was employed to detect Cd(II) in tobacco samples and environmental water samples such as wastewater, bottled water, well water, and spring water from different sources. As given in Table 4, the Cd(II) concentration was found to be 7.94 nM in wastewater while it was not detected in the remaining three water samples. Cd(II) was found to be 16.1 ng g^−1^ in tobacco samples. The recovery of Cd(II) in the samples was determined by using standard addition method and it was between 96.00% and 105.2%, which shows the good recovery of the method.

Graphite furnace atomic absorption spectroscopic (GFAAS) technique was also used to the determination of Cd(II) in the given samples for the comparison purpose. The results found from the two methods were similar and acceptable (Table 4). Paired *t* test with a 95% confidence limit (*p* = 0.05) was used to check the significant similarity or difference of the results from the methods. The *t* test proved that there is no significant difference in the Cd(II) concentrations by the two methods at a 95% confidence limit. The results confirm the applicability of the developed electrode for accurate and reliable quantification of Cd(II) in the real samples.

## 4. Conclusion

In this work, a novel, simple, highly sensitive, and selective CPE modified with HDPBA has been fabricated for the detection of Cd(II) in several environmental water samples and tobacco samples. The developed electrode has many attractive features including low cost, simple preparation, easy renewable ability, portability, and low background noise. Furthermore, the proposed electrode showed various interesting electrochemical behaviors such as excellent selectivity, high sensitivity, good stability, high speed, and capability for the quantification of Cd(II) at very low concentrations compared to unmodified CPE and most other reported electrodes in the literature.

Under optimized experimental parameters, the developed electrode displayed a wide linear range (0.3–100 nM), a very low detection limit (0.032 nM Cd(II)), and good reproducibility (RSD of 3.8%). The developed method and the GFAAS method were effectively employed for the determination of Cd(II) in tobacco and water samples. The recoveries obtained from the two methods were compared, and it was found that they had good agreement. Even though the developed method was applied to the determination of Cd(II) in tobacco and different types of water samples, it can also be employed for Cd(II) determination in other matrices.

## Figures and Tables

**Table 1:** Influence of modifier (HDPBA) content in CPE on the current response of Cd(II).

S.N.	HDPBA content in CPE (% w/w)	Peak current of Cd(II) (*µ*A)
1	2.0	61.85
2	3.5	107.7
3	5.0	216.3
4	7.5	182.4
5	10.0	150.6
6	12.5	104.3

**Table 2:** Change in current response of 5.0 × 10^−8^ M Cd(II) in the presence of other ions.

Interfering ion	Concentration (M)	Change of current (%) of Cd(II)
K^+^, Na^+^, Ca^2+^, Mg^2+^, NH_4_^+^, Cl^−^ and NO_3_^−^	5.0 × 10^−6^	4.51
Ni^2+^ and SO_4_^2-^	1.5 × 10^−6^	3.96
Fe^3+^, Hg^2+^ and Co^2+^	1.0 × 10^−6^	−4.34
Pb^2+^	5.0 × 10^−7^	−3.60
Cu^2+^	3.5 × 10^−7^	−4.24

**Table 3:** Comparison of the performance of HDPBA modified CPE with other reported sensors for the determination of Cd(II).

Electrode^a^	Technique	Accumulation time (s)	Linear range (nM)	LOD (nM)	Ref.
CS-MCPE	DPASV	600	1780–5785	934	[34]
MWCNT-MES-CPE	DPASV	500	27–2225	21.4	[39]
PrGO/AuNPs/Sal-Cys/GCE	SWASV	120	1–10	0.060	[40]
Cr-CPE	SWASV	100	89–7120	26.7	[41]
L/MWCNTs/CPE_IL_	DPASV	90	1.8–205.0	0.700	[18]
N-BDMP-CPE	SWASV	210	89–17800	58.7	[42]
Bi/MCNTs-CPE	SWASV	200	8.9–534.0	2.67	[43]
PPy-CO_2_@PGE	DPASV	900	0.1–1.0	0.023	[44]
Nafion-G/GCE	DPASV	120	13.3–267.0	0.178	[45]
Ligand L/CPE	DPV	—	17.8–89	0.466	[46]
Fe_3_O_4_@G2-PAD	SWASV		4.4–711	1.867	[3]
HDPBA-CPE	SWASV	270	0.3–100	0.032	Present work

^a^CS: coconut shell powder; MES: magnetic nanocomposite of Fe_3_O_4_/eggshell; PrGO/AuNPs/Sal-Cys: penetrable nature of graphene/gold nanoparticles/modified L-cysteine nanocomposite; Cr: chromium (III) oxide; L/MWCNTs/CPE_IL_: 4-((1H-1,4-triazol-3-ylimino)methyl)phenol (L)-MWCNTs composite modified carbon paste ionic liquid electrode; N-BDMP: nitro benzoyl diphenyl methylene phosphorane; Bi/MCNTs: bismuth modified multiwalled carbon nanotubes doped; PPy-CO_2_@PGE: a pencil graphite electrode modified with pyrrole-1-carboxylic acid; Nafion-G: Nafion-graphene composite film; Ligand L/CP: 2,2'-((pyridine-2,6-diylbis (azanylylidene))bis(methany-alkylidene)) bis(4-bromo-phenol); and Fe_3_O_4_@G2-PAD: second-generation polyamidoamine dendrimer functionalized magnetic nanoparticles.

**Table 4:** Results of determination of Cd(II) in the real samples using the developed method (*n* = 5) and GFAAS method.

Sample	Added (nM)	SWASV method detected (nM)	SWASV method recovery (%)	GFAAS method detected (nM)	GFAAS method recovery (%)
Wastewater	0	7.94 (±0.13)	—	8.09 (±0.21)	—
	30	39.5 (±0.6)	105.2	39.3 (±0.8)	104.0

Bottled water	0	ND	—	ND	—
	30	30.8 (±0.7)	102.7	31.3 (±0.4)	104.3

Well water	0	ND	—	ND	—
	30	29.8 (±0.5)	99.33	29.6 (±0.7)	98.66

Spring water	0	ND	—	ND	‒
	30	28.8 (±0.2)	96.00	29.5 (±0.4)	98.33
	(ng·g^−1^)	(ng·g^−1^)		(ng·g^−1^)	

Tobacco	0	16.1 (±0.2)	—	15.9 (±0.1)	—
	30	47.2 (±1.1)	103.7	46.6 (±1.2)	102.3

ND, not detected.

## Data Availability

All data analyzed or generated in this research work are included in this paper. The authors declare that there are no additional data with the authors.
